# Edible Mushrooms: A Comprehensive Review on Bioactive Compounds with Health Benefits and Processing Aspects

**DOI:** 10.3390/foods10122996

**Published:** 2021-12-04

**Authors:** Krishan Kumar, Rahul Mehra, Raquel P. F. Guiné, Maria João Lima, Naveen Kumar, Ravinder Kaushik, Naseer Ahmed, Ajar Nath Yadav, Harish Kumar

**Affiliations:** 1 Department of Food Technology, Dr. Khem Singh Gill Akal College of Agriculture, Eternal University, Baru Sahib, Sirmaur 173101, Himachal Pradesh, India; krishankumar02007@gmail.com (K.K.); drnaseerahmed@eternaluniversity.edu.in (N.A.); ajar@eternaluniversity.edu.in (A.N.Y.); 2 Amity Institute of Biotechnology, Amity University Rajasthan, Jaipur 303002, Rajasthan, India; rahulmehranov@gmail.com (R.M.); nkft87@gmail.com (N.K.); 3 CERNAS Research Centre, Polytechnic Institute of Viseu, 3504-510 Viseu, Portugal; mjoaolima@esav.ipv.pt; 4 School of Health Sciences, University of Petroleum and Energy Studies, Dehradun 248001, Uttrakhand, India; ravinder_foodtech2007@rediffmail.com

**Keywords:** bioactive components, edible mushrooms, health benefits, nutraceutical’s potentials

## Abstract

Mushrooms are well-known functional foods due to the presence of a huge quantity of nutraceutical components. These are well recognized for their nutritional importance such as high protein, low fat, and low energy contents. These are rich in minerals such as iron, phosphorus, as well as in vitamins like riboflavin, thiamine, ergosterol, niacin, and ascorbic acid. They also contain bioactive constituents like secondary metabolites (terpenoids, acids, alkaloids, sesquiterpenes, polyphenolic compounds, lactones, sterols, nucleotide analogues, vitamins, and metal chelating agents) and polysaccharides chiefly *β*-glucans and glycoproteins. Due to the occurrence of biologically active substances, mushrooms can serve as hepatoprotective, immune-potentiating, anti-cancer, anti-viral, and hypocholesterolemic agents. They have great potential to prevent cardiovascular diseases due to their low fat and high fiber contents, as well as being foremost sources of natural antioxidants useful in reducing oxidative damages. However, mushrooms remained underutilized, despite their wide nutritional and bioactive potential. Novel green techniques are being explored for the extraction of bioactive components from edible mushrooms. The current review is intended to deliberate the nutraceutical potential of mushrooms, therapeutic properties, bioactive compounds, health benefits, and processing aspects of edible mushrooms for maintenance, and promotion of a healthy lifestyle.

## 1. Introduction

Mushrooms are believed to be the main underutilized resource of nutritious foods. Their cultivation is, at present, the most cost-effective biotechnology for the transformation of lignocelluloses waste into protein-rich foods, besides causing a considerable reduction in environmental pollution [1]. There are around 1600 species of mushrooms, however only 100 species have been recognized to be consumed for edible purposes. About 33 species of edible mushrooms are under cultivation throughout the world, but only three species are commonly grown, i.e., white button mushrooms (*Agaricus bisporus* L.), oyster mushroom (*Pleurotus ostreatus* L.), and paddy straw mushroom (*Volvariella volvacea* L.) [2]. These were incorporated into the diet by Romans and Greeks since early times. They were considered as food of God by Romans, while the Chinese designated them as an elixir of life [3]. Many cultures have utilized them for centuries. These are rich sources of fiber, and nutrients such as proteins, minerals, vitamins with lower amounts of fats and calories [4].

Edible mushrooms are generally used as a source for the preparation of nutraceuticals and drugs with anti-tumour, antioxidant, and antimicrobial properties. In addition to their pharmaceutical properties, mushrooms are also essential in our diet, due to their low fat content, high protein, and low energy contents [5]. The mushroom proteins comprise all essential amino acids mandatory for humans. Besides, these comprise many nutritional components such as iron, phosphorus, and vitamins like ascorbic acid, thiamine, riboflavin, niacin, and ergosterol [6].

Mushrooms are priced for texture, flavour, and some therapeutic characteristics. Various studies have also reported that, due to the occurrence of functional components in mushrooms, these have antiviral, antitumor, antithrombotic, and immuno-modulating characteristics [7]. The polysaccharides derived from edible mushrooms, especially β-glucans, are gaining the attention of scientists and other food industries due to their antioxidant, antidiabetic, anticarcinogenic and immune-modulating effects as well as other health benefits. There are numerous conventional and advanced extraction techniques employed nowadays for the recovery of bioactives from mushrooms. The extraction techniques such as microwave-assisted, ultrasound-assisted, enzyme-assisted, subcritical water, pulsed electric field-assisted, and integrated extraction are novel methods for extracting bioactive components [8]. Edible mushrooms are becoming more popular as health promoters, and have led to advancements in the research activities focused on different types of mushrooms. These can have broad applications to supplement various staple food products, due to their capability to improve protein content, along with the valued health benefits of bioactive compounds. Although a lot of information is available in literature on bioactive components of edible mushrooms as well as their health benefits, but information on techniques for extraction of bioactives from edible mushrooms as well as the processing aspects of edible mushrooms is scanty in literature. The objective of this review is to compile existing facts about the bioactive components, their nutraceuticals potential, use of conventional and novel techniques for extraction of bioactive components, and the processing aspects of edible mushrooms.

## 2. Bioactive Components in Edible Mushrooms

**Figure 1:**
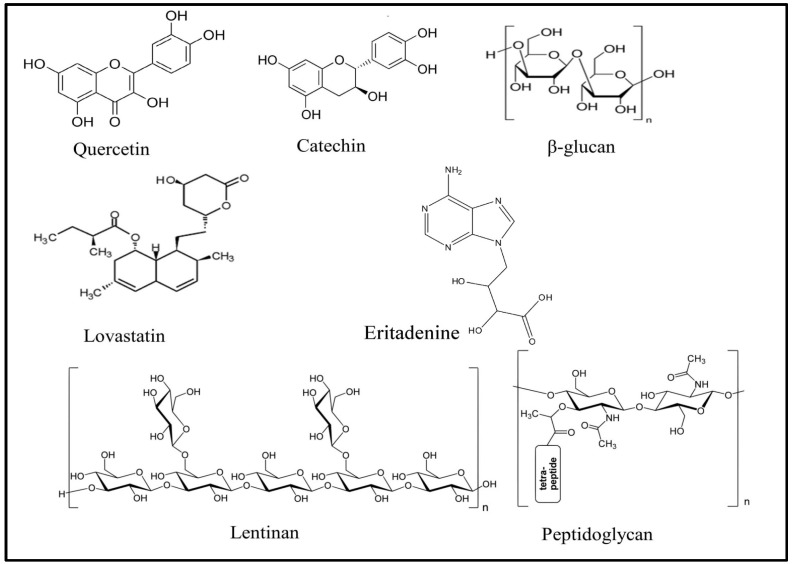
Bioactive components reported in different edible mushrooms.

Bioactive components present in the cell wall polysaccharides of mushrooms have numerous functional characteristics, specifically anti-tumour, immune-stimulating, antioxidant and hypoglycemic effects, as described in many in vitro as well as in vivo studies. However, the detailed mechanism of their effect is not yet completely explored [9,10,11,12,13]. Bioactive components, present in different types of mushrooms, and their health benefits are depicted in Table 1, and the chemical structure of some common bioactive compounds found in different mushrooms is presented in Figure 1.

Mushrooms produce different types of bioactive components, like phenolics, terpenoids, polysaccharides, glucans, and lectins, which are reported to put forth more than 126 health benefitting effects, including anti-microbial, immune-modulating, antioxidant, antiviral, and hypo-cholesterolemic [39]. Biomolecules, such as terpenoids and glucans with immuno-modulating and antimicrobial characteristics, can be extracted from mushrooms dwelling on different types of wood belonging to *genera Fomes, Ganoderma, Phellinus, Trametes Fomitopsis, Inonotus*, and *Schyzophillum* [40]. Proteins present in different types of mushrooms possess numerous biological characteristics, including lectins, lignocellulose-degrading enzymes, proteases, ribosome-inactivating proteins, protease inhibitors, and hydrophobins, showing great potential to be utilized in different biotechnological applications for the preparation of new drugs [41]. Erbiai et al. [2] determined the chemical composition, bioactive compounds, and antioxidant activity of two wild edible mushrooms; the honey fungus (*Armillaria mellea*) and the parasol mushroom (*Macrolepiota procera*), collected from Northern Morocco and Portugal. The methanolic extracts showed a strong DPPH free radical-scavenging activity (IC50 1.06–1.32 mg/mL). The mushroom species with the highest antioxidant capacity was *A. mellea* from Northern Morocco. LC-MS analysis of individual phenolic compounds revealed that vanillic acid (198.40 µg/g dry weight (dw) and cinnamic acid (155.20 ± 0.97 µg/g dw) were the main compounds detected in *A. mellea*, while protocatechuic acid (92.52 and 125.50 µg/g dw) was predominated in *M. procera* for Northern Morocco and Portugal samples, respectively.

Extracts from medicinal mushrooms like *Polyporus umbellatus* and *Polyporus alveolaris* containing different types of polypeptides and cytotoxic steroids were found to possess immuno-stimulating, anti-cancer, anti-inflammatory, hepatoprotective, anti-fungal, and anti-bacterial effects [42]. Oyster mushrooms (*Pleurotus* species) have hypocholesterolemic, antioxidant, anti-bacterial, anti-diabetic, hepatoprotective, anti-carcinogenic, anti-viral, anti-arthritic, and immune-modulatory properties. Protein deficiency, particularly in developing countries, due to the unacceptability of animal proteins because of religious obligations, can be overcome using these edible mushrooms [43]. Barros et al. [44] estimated different types of bioactive components such as fatty acids by GC-FID, tocopherols by HPLC-fluorescence, and spectrophotometric methods to evaluate flavonoids, phenolics, carotenoids, and ascorbic acid. They studied the anti-microbial potential of the mushrooms, and these were found effective against Gram-positive and Gram-negative bacteria and fungi.

## 3. Techniques Involved in Extraction of Bioactive Components from Edible Mushrooms

Mushrooms are rich sources of nutritionally important components, like proteins, polysaccharides, lipids, polyphenolic components, vitamins, and other micronutrients.

Mushroom processing industries produce a diverse range of by-products during canning, pickling, and processing. Organic wastes generated by these sectors are hazardous to the environment and have the potential to be exploited as a bioresource for extracting bioactives from edible mushrooms. The extracted bioactive chemicals can be employed in the food and pharmaceutical sectors as nutraceuticals and nutritional supplements.

In particular, they are rich in B-group vitamins and can be a good source of these vitamins for vegetarians. The extraction of bioactive components from mushrooms using water or organic solvents can result in the degradation of these valuable compounds [45].

### 3.1. Conventional Techniques Used in Extraction of Bioactive Components

Conventional techniques use organic solvents or water for extracting bioactives from various bioresources. Solvent-assisted extraction is a traditional method used for extracting valuable compounds from mushrooms [46]. Extraction using water is also a commonly used technique, as it is economical and does not need any special equipment. Still, it needs high temperatures ranging from 50–80 °C [47,48], and is time-consuming, requiring about 1.5–5 h [48,49]. The high temperature and long duration results in the degradation of thermolabile components present in mushrooms. Hydro-alcoholic extractions necessitate an adequate temperature of 25–60 °C, for a time ranging from 1–24 h, as well as high concentrations (30 to 98.6%) of high-cost organic solvents [48,49]. Extraction using organic solvents such as chloroform-methanol 2:1 (*v*/*v*), percolation, maceration (soaking), pressurized liquid extraction, and the Soxhlet method is widely used to extract nutraceutical components from plant bioresources. Conventional extraction techniques generally involve the usage of vast quantities of solvents, and they can cause the degradation of heat-labile components [50]. Therefore, the search for novel sustainable extraction techniques has been increasing by food industries for extraction of valuable compounds from mushrooms.

### 3.2. Use of Novel Extraction Techniques

There are numerous novel techniques used for the extraction of bioactive components from the plant, as well as animal bioresources, which include enzyme assisted extraction, subcritical and supercritical fluid extraction, extraction using a pulsed electric field, ultrasound-assisted extraction, microwave-assisted extraction, and subcritical water extraction. Each category of extraction technique has a specific area of application, so a brief review of these extraction techniques is discussed below.

#### 3.2.1. Enzyme-Assisted Extraction

This technique involves processing at a lower temperature for a short time, lower consumption of energy, and higher yield in the food processing industry. The cell wall of edible mushrooms contains polysaccharides like chitin and glucans which can be hydrolysed by using hydrolytic enzymes such as glucanase or chitinase [51]. Therefore, the use of enzymes for the hydrolysis of polymers present in the cell walls of mushrooms can help to improve the extraction of bioactive components. The mushroom *Trichoderma harzianum* is widely used for the extraction of eritadenine, a bioactive component present in mushrooms [52].

#### 3.2.2. Supercritical and Subcritical Fluid Extraction

In recent years, conventional extraction techniques are being replaced by subcritical as well as supercritical fluid extraction. These have attracted the attention of scientists because these are environment-friendly techniques, and can provide higher extraction yields of bioactive from different food sources, including mushrooms in comparison to classical extraction techniques [53]. Any substance that is subjected to pressure and temperature higher than its critical point, where distinct gas and liquid phases do not exist, is termed as a supercritical fluid. Their fluid properties can be placed between gas and liquid. A supercritical fluid has a density equivalent to any liquid and viscosity just like a gas, but its diffusivity lies between the liquid and gas phases, thus assisting the extraction of intracellular components [54,55].

Vidović et al. [56] extracted fatty acids using subcritical and supercritical carbon dioxide extraction method from *Boletus edulis* mushroom. The pressure and extraction time had a significant effect on the extraction yield in both extraction processes. Higher extraction yields have been obtained by subcritical carbon dioxide, and higher linoleic acid content has been determined in extracts obtained by supercritical carbon dioxide. Seo and Lee [57] utilized the subcritical water extraction (SWE) technique to recover bioactive compounds from golden oyster mushroom with high temperatures (50–300 °C) and different pressures (0.002–5 MPa), and it was revealed that extracts possessed higher antioxidant activities when higher temperatures (250–300 °C) and longer times were used. Similarly, Yang et al. [58] extracted bioactives from *Grifola frondosa* using a subcritical water extraction technique at a temperature of 210 °C for 43.65 min. The extracts obtained after SWE yielded twice as many polysaccharides as traditional hot water extraction.

#### 3.2.3. Ultrasound-Assisted Extraction (UAE)

Ultrasound-assisted extraction has wide applications and has attracted the attention of food scientists due to their wide benefits in the retrieval of value-added compounds from different sources [59,60]. In this process, cells of mushrooms are disrupted by cavitation bubbles, thereby facilitating the mass transfer and increasing the extraction yield. Besides, the recovery of important bioactive compounds from plant sources using ultrasounds is a low-cost technique. The temperature and extraction time can be lowered by combining this technique with solvent extraction, and it can help in the preservation of heat-sensitive components [61]. It has been extensively used by scientists to extract nutritional components from mushrooms [62,63]. Cheung et al. [62] utilized the UAE technique for the extraction of bioactive polysaccharides from the fruit body and fungal mycelia of mushrooms, such as *Grifola frondosa* and *Lentinus edodes*. The total yield of the extract was about 55% after extraction for 60 min. The polysaccharides extracted from *Grifola frondosa* have amounted to 0.05%, and that from *Lentinus edodes* was 0.13%. Whereas, You et al. [64] extracted 8.21% of bioactive trametes orientalis from *Boletus edulis*.

#### 3.2.4. Extraction Using Pulsed Electric Fields

Pulsed electric fields (PEF) have been widely used over several years for intracellular extraction of bioactive components from plant food materials [65,66], agricultural by-products [65,67], and several bio-suspensions [68,69], owing to their capability to cause lethal injury to the cells by the temporary permeabilization of cell membranes. Moreover, the extraction of bioactive components is increased due to the movement of charged particles between various parts of the cell. Xue and Farid [70] studied the effect of continuous PEF treatment on the extraction of white button mushroom suspension (9% *w*/*w*), with pulsed electric field intensity in the range of 12.4 to 38.4 kV/cm and bipolar square pulses of 2 μs pulse duration. The optimum extraction yields 98% (7.9 mg/g mushroom) of polysaccharide, 51% (1.6 mg GAE/g mushroom) of total polyphenolic components, and 49% (2.7 mg/g of mushroom) proteins. Parniakov et al. [71] compared the efficiency of extraction and stability of extracts from *Agaricus bisporus* for different methods of extraction, i.e., pressure extraction (PE), pressure extraction assisted by pulsed electric field (PE  +  PEF), hot water extraction (WE), ethanol extraction (EE), and supplementary ethanol extraction. They reported that the extracts, produced by PE and PE  +  PEF methods, were clear, and their colloid stability was high. The PE  +  PEF method gave higher nucleic acid/proteins ratio as compared with that of PE method. Moreover, PE  +  PEF method produced mushroom extracts with high contents of fresh-like proteins and polysaccharides.

#### 3.2.5. Extraction Using Microwaves

This process involves the heating of food material and evaporation of moisture, which can help to generate great pressure as well as rupture of cells, thereby assisting the discharge of the required components from plant cells [72]. Microwaves have their electromagnetic spectrum ranging between frequencies of far infrared light and radio waves. Their frequency varies from 300 MHz to 300 GHz; for industrial as well as scientific purposes, 915 and 2450 MHz are the most commonly applied microwave heating frequencies [73]. The microwaves cause a heating effect in food materials based on the dielectric properties of materials. The efficiency of transforming electromagnetic radiation into heat energy is measured by the dielectric loss [74]. Lebovka et al. [65] extracted bioactive from Clitocybe maxima using the MAE technique. The total yield of polysaccharides was 9.24%, which was 57.8% higher than the water extraction technique. Similarly, Özyürek et al. [75] extracted bioactive from Boletus edulis, *Terfezia boudieri Chatin*, and *Lactarius* volumes using 80% methanol at a temperature of 80 °C with an extraction time of 5 min. The methanolic extracts obtained after using MAE were highly effective among other extraction techniques.

#### 3.2.6. Subcritical Water Extraction

This technique utilizes hot water at a temperature ranging from 100 to 374 °C (the latter is the critical temperature of water) under high pressure to keep water in the liquid state. It is termed hot pressurized water extraction [76]. Compounds with high polarity can be easily dissolved in water at low temperatures, whereas super-heated water (having a temperature above 100 °C) can serve as an organic solvent. Smith [77] extracted bioactive compounds from *Ganoderma lucidum* using a subcritical water extraction technique. He reported a yield of 328 mg water-soluble organic compounds per g dried sample at 200 °C/60 min by the batch processing method. Moreover, he extracted 0.44 mg β-glucan/100 g of sample and 241 mg water-soluble organic compounds per g of sample dried at 200 °C, during extraction by batch processing system for 130 min.

## 4. Health Benefits of Bioactive Components Present in the Mushroom

**Figure 2:**
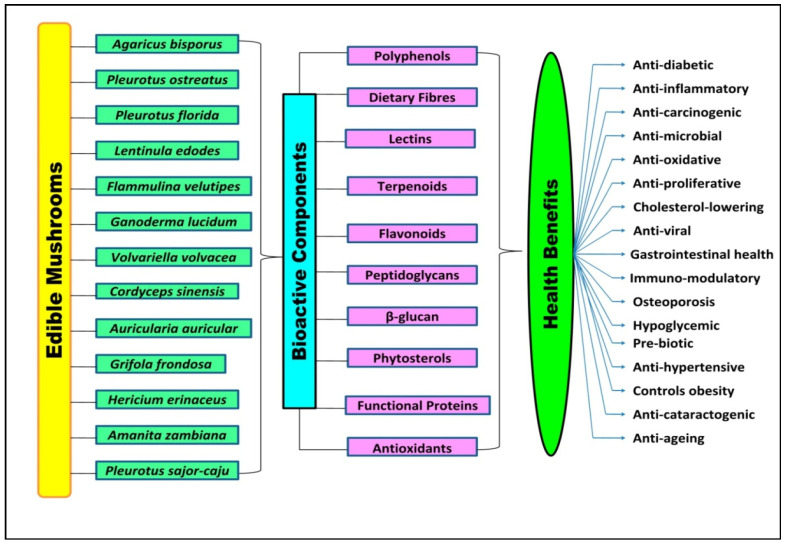
Edible mushrooms, bioactive components, and health benefits.

Edible mushrooms have numerous therapeutic properties due to the presence of a large amount of bioactive and nutraceutical components. These are considered highly effective against various lifestyle diseases such as liver diseases, cancer, diabetes, and cardiovascular diseases [78]. Furthermore, antioxidants and anti-microbial agents make them able to have immuno-modulatory, anti-ageing, and anti-microbial effects (Figure 2). Various health benefits of edible mushrooms are discussed under the following subheadings.

### 4.1. Anti-Carcinogenic Properties

Medicinal mushrooms contain numerous bioactive substances with potential anticancer properties. These compounds comprise dietary fibre, polysaccharides, complexes of polysaccharides and proteins, steroids, terpenoids, phenolics, and certain types of proteins. Daba and Ezeronye [79] utilized diverse cancer cell lines to discover the anti-tumour effect of fruit bodies as well as mycelial extracts of mushrooms. Polysaccharides extracted from mushrooms expressed potential anti-tumour activity against mammary adenocarcinoma 755, sarcoma 180, and leukaemia L-1210. Patel and Goyal [80] reported that the mushrooms possessing anti-carcinogenic characteristics are from genus *Pleurotus, Phellinus, Agaricus, Clitocybe, Ganoderma, Trametes, Antrodia, Xerocomus, Cordyceps, Schizophyllum, Calvatia, Flammulina, Inonotus, Suillus, Albatrellus, Inocybe, Funlia, Russula, Lactarius*, and *Fomes*. Compounds present in mushrooms having anti-cancer characteristics which play an important role as reactive oxygen species (ROS) inducer, anti-mitotic, a mitotic kinase inhibitor, topoisomerase inhibitor, and inhibition of angiogenesis causing apoptosis of cancer cells, ultimately preventing cancer proliferation.

Baker et al. [81] reported anti-tumour, immune-modulating, and anti-metastasis properties in *Phellinus linteus*. Aqueous extract of polysaccharide from *Pleurotus ostreatus* possesses pro-apoptotic and anti-proliferative effects on HT-29 cells of colon cancer [82]. Moreover, a polysaccharide extracted from *Agaricus blazei* suppressed angiogenesis in-vivo, thereby proving the anti-carcinogenic potential of polysaccharides extracted from edible mushrooms [83]. Mushrooms having medicinal characteristics from south India such as *Pleurotus pulmonaris, Phellinus rimosus, Pleurotus florida*, and *Ganoderma lucidum* were found to have profound antioxidant and anti-tumour activities. These are reported to be valuable sources of antitumor and antioxidant components, and possess potential anti-carcinogenic and anti-mutagenic activities [84]. Button mushrooms (*Agaricus bisporus* L.) are found to have great potential for reducing breast cancer, because of their capacity to diminish aromatase activity and biosynthesis of oestrogen, as reported by in-vivo and in vitro studies [85].

Shin et al. [86] exposed the relationship of mushrooms intake in decreasing the risk of breast cancer in 358 female and 360 cancer-free (control) women in Korea and concluded that higher consumption of mushrooms was linked with lesser risks of breast cancers amongst premenopausal women. They further observed that this association may be stouter in women suffering from hormone receptor-positive tumours. Daba and Ezeronye [79] evaluated the anti-tumour activity of mycelial extracts and mushroom fruit bodies by diverse cancer cell lines.

### 4.2. Anti-Oxidative Properties

Antioxidant components present in different food types have the competence to entrap free radicals and inhibit the oxidative changes responsible for causing different types of degenerative diseases [87]. Natural antioxidants are present in whole grains, vegetables, fruits, spices, tea, and herbs. Mushrooms, owing to phenolic components and other polysaccharides, have also been described as a rich source of antioxidant components [88,89,90]. Dietary supplementation of edible mushrooms can reduce oxidative stress by increasing antioxidant defences. Mushrooms, either cultivated or wild, have substantial antioxidant characteristics, mainly due to bioactive components such as polyphenolic compounds, carotenoids, polysaccharides, and vitamins. Owing to the occurrence of antioxidants and other health-promoting components, edible mushrooms are used as prevalent delicacy foods [91].

Liu et al. [92] demonstrated the in vitro free radical scavenging activity of mushroom cell wall polysaccharides, and stated that these have great antioxidant potential. Chowdhury et al. [25] prepared the methanolic extract by mixing a fine-dried mushroom powder sample (100 g) by stirring with 100 mL of methanol at 25 °C and 150 rpm for 24 h, and filtered through Whatman no. 4 paper. After analysing the extract, we observed that the total phenolic components are the main bioactive compounds in extracts of mushrooms fruiting bodies, and expressed as mg GAE/g of the fruiting body. Phenolic components were found to vary from 3.2–10.7 mg GAE/g. Flavonoid contents ranged from 2.5 mg/mL–4.8 mg/mL of extract; and vitamin C varied from 0.06 mg/mL to 0.21 mg/mL. Therefore, all the isolates contained high phenol as well as flavonoid contents, except oyster mushroom (*Pleurotus ostreatus*), but contents of ascorbic acid were found in very small amounts. Kosanic et al. [93] estimated the in vitro antioxidant activity of the methanol and acetone extracts of some edible mushrooms such as *C. cibarius, A. rubescens,* L. *apparatus*, and *R. cyanoxantha*. They concluded that acetone extract of *Russula cyanoxantha* mushrooms possessed the maximum antioxidant activity as compared to other extracts. Therefore, the use of mushrooms in the daily diet as sources of natural antioxidants might be advantageous for the prevention or reduction of oxidative damage and other lifestyle diseases.

### 4.3. Hypo-Cholesterolemic Agents

Cardiovascular disorders are linked with hypercholesterolemia, low-density lipophilic oxidation, and atherosclerosis. Therefore, blood cholesterol level needs to be regulated for the prevention as well as treatment of this disease. The low fat and high fibre contents of edible mushrooms make them the best food for the prevention of cardiac ailments. Supplementation of edible mushrooms is a natural hypo-cholesteromic and an anti-sclerotic diet that is regularly recommended in oriental medicine [94]. It has been observed that the utilization of *Termitomyces microcarpus* mushrooms has significantly reduced the occurrence of diseases related to high blood lipids, and further suggested that high quantities of fibre in the mushrooms can reduce total serum cholesterol, LDL-cholesterol, and triglycerides [95].

Exo-polymers synthesized in the submerged culture of *Auricularia auricula-judae, Hericium erinaceus, Phellinus pini, Grifola frondosa* (GF) and *Flammulina velutipes* have a hypolipidemic effect on the experimental animals [96]. Rathee et al. [97] isolated and identified an active hypo-cholesterolemic component, eritadenine [2(R), 3(R)-dihydroxy-4-(9-adenyl)-butyric acid], in the shiitake mushroom. The eritadenine can decrease the level of serum cholesterol in mice by speeding up the elimination of consumed cholesterol, as well as its metabolic decomposition.

### 4.4. Hepatoprotective Effects

Damage to the liver is caused mainly by oxidative stress, and is characterized by fibrosis, chronic hepatitis, hepatocellular carcinoma, and cirrhosis [98]. An injury produced in the liver with diminished liver function is known as hepatotoxicity, and can be caused by the intake of any drug or different non-infectious agents [99]. Similarly, the ethanolic extract from *Calocybe indica* has been reported as having a the positive impact against hepatic injury caused due to carbon tetrachloride poisoning in mice and concluded that this extract protected against CCl4 induced hepatotoxicity [100]. Ganosporeric acid A and ganoderic acids R and S extracted from *Ganoderma lucidum* possessed in vitro anti-hepatotoxic effects in primary cultured rat hepatocytes when examined with the galactosamine-induced cytotoxic test [101]. Extracts from the basidiomass of *Ganoderma frondosa* and *Lentinus edodes* (100 mg/kg of body weight) were found to be highly effective in decreasing the paracetamol-induced proliferation of alanine transaminase and aspartate transaminase levels, whereas the mycelial aqueous extracts from *Tricholoma lobayense* showed hepatoprotective effects at higher doses of 300 mg/kg of body weight [102]. Sumy et al. [103] studied the hepatoprotective effects of *Pleurotus florida* against the injury caused due to the intake of paracetamol in albino rats. The experimental results exposed profound antioxidants, as well as hepatoprotective effects of ethanolic extract of the *Morchella esculenta mycelium*.

*Ganoderma lucidum* is an extensively studied variety of mushrooms with prevalent hepatoprotective effects. About 400 chemical substances separated from *Ganoderma lucidum,* such as tri-terpenoids, polysaccharides, ergo-sterols, nucleosides, proteins, peptides, fatty acids, and trace elements, have hepatoprotective effects. Out of these, triterpenoid and polysaccharides were found as potential bioactive constituents, with a significant protective effect against liver injury caused by various toxins [104]. Triterpenoids can inhibit the activity of β-glucuronidase, an indicator of the intensity of liver damage [105]. Insoluble non-starch polysaccharides extracted from the mycelium of *Pleurotus ostreatus* prevented hepatic damage in rats, caused due to carbon tetrachloride poisoning. Hepatic injury caused due to thioacetamide poisoning in mice can be prevented using polysaccharopeptides extracted from *Pleurotus ostreatus* [106]. Polysaccharides extracted from *Hericium erinaceus* can be utilized as a supplement for the prevention of various liver diseases [107]. The polysaccharide-rich extract of *Pleurotus eryngii* has been discovered to have hepatoprotective as well as hypolipidemic effects, and can be utilized as an indispensable functional food additive [108]. Similar activity has been shown by extracts from *Agaricus blazei* against paracetamol-induced liver injury [109].

### 4.5. Anti-Diabetic Effects

Diabetes mellitus is a metabolic problem that can be controlled with an improved standard of living, exercise, and a suitable diet. Mushrooms can serve as functional foods in controlling diabetes. These are excellent sources of bioactive components with anti-diabetic properties. Many species of mushrooms are highly effective in controlling blood glucose levels and diabetic difficulties. Mushrooms like *Agaricus subrufescens, Agaricus bisporus, Coprinus comatus, Cordyceps sinensis, Inonotus obliquus, Ganoderma lucidum, Pleurotus *spp.*, Phellinus linteus, Sparassis crispa*, and *Poria cocos* are reported in various studies to exert hypoglycemic effects [110]. The edible mushrooms contain a very low amount of fat, cholesterol, carbohydrates, and are rich in protein, vitamin, and mineral contents, and thus are considered as low-calorie foods for diabetic patients [110,111].

Kaur et al. [112] described mushrooms as the best sources of natural medications with anti-diabetic characteristics. These are considered functional foods and a significant source of bioactive compounds, such as proteins, lipids, and polysaccharides, as well as metabolites such as alkaloids, terpenoids, lactones, lectins, sterols, and phenolic components, with high therapeutic activities. Cho et al. [113] discovered anti-diabetic activities in the exopolysaccharides generated in the submerged culture of *Tremella fuciformis* in ob/ob mice. Rushita et al. [114] studied the hypoglycemic characteristics of methanolic extract from *Pleurotus citrinopileatus* against streptozotocin-induced diabetes mellitus (type-2) in rats. There was a substantial decline in the fasting level of blood glucose, as well as the activity of serum catalase, but a significant surge in level of serum insulin was observed in groups treated with high dose of mushroom extract as compared to the untreated group.

β-Glucan, an important polysaccharide, is widely present in mushrooms. It has been found to repair the activities of pancreatic tissues by enhancing the secretion of insulin by β-cells, leading to decreased levels of blood glucose. Lectins isolated from *Agaricus campestris* and *Agaricus bisporus* increased the release of the hormone insulin from islets of Langerhans in the pancreatic tissues of rats [115]. The ethanolic extract of *Pleurotus ostreatus* caused a significant reduction in the serum glucose level of alloxan-induced diabetic mice. The level of urea and creatinine in serum decreased significantly in the post-treated groups. It was found that *Pleurotus ostreatus* can be utilized in medicinal preparations useful in diabetes mellitus [116].

### 4.6. Anti-Microbial Effects

Mushrooms are considered as the best nutritional supplements, with outstanding medicinal values. Certain edible mushrooms have antimicrobial properties and can control various human diseases. These were found to have anti-fungal and anti-bacterial activities against resilient disease-causing microbes [117]. The presence of phenolic compounds in *Inonotus hispidus* and ergosterol peroxide in numerous mushrooms was found to exert in vitro anti-viral effects against influenza viruses [118].

Chowdhury et al. [25] discovered anti-microbial activities in some varieties of edible mushrooms in Bangladesh. The zone of inhibition varied from 7 to 20 mm against all fungi and bacteria. The best antimicrobial activity was reported in *Lentinula edodes* as compared to other mushroom varieties. *Pleurotus aeruginosa* was moderately resistant and *Saccharomyces cerevisiae* was more sensitive as compared to other microbial isolates.

Chen and Huang [119] screened the culture filtrates from 27 eatable mushrooms for anti-microbial activities. The filtrates of *Clitocybe nuda* and *Lentinula edodes* were found to completely prevent the germination of spores in *Colletotrichum higginsianum*. Culture filtrates from three mushrooms, i.e., *Ganoderma lucidum, Lentinus edodes*, and *Clitocybe nuda* could entirely obstruct the germination of spores in *Alternaria brassicicola*. Therefore, the bioactive components from mushrooms have the prospective to be established into biocontrol agents against various plant diseases. Methanol and acetone extracts of the mushrooms *Cantharellus cibarius, Amanita rubescens, Russula cyanoxantha*, and *Lactarius piperatus* were found to have in vitro antimicrobial activity [93].

Menaga et al. [30] reported that bioactive components extracted from *Pleurotus florida* can be employed as alternative therapeutics such as antibiotics. Similarly, Alves et al. [120] found *Russula delica, Fistulina hepatica,* and *Russula botrytis* to be the most capable anti-microbial agents. It concluded that mushrooms can also be used for pharmaceutical purposes in the treatment of several diseases. Shen et al. [121] reported that mushroom extracts can be utilized as food additives with antioxidant and antimicrobial activity to encounter the growing demands for food quality and safety, thereby preventing the spoilage of food products.

### 4.7. Mushrooms as Natural Resources of Immunotherapy

Mushrooms are well-known as significant natural sources of immunotherapeutic components. These can be utilized as immune-stimulating and immune-modulating agents in treating certain immunodeficiency maladies such as cancer, tumour, HIV, and tuberculosis. Bioactive components extracted from *Pleurotus* mushroom are capable of enhancing or balancing an immune response in the human body. Such bioactive components include polysaccharide-proteins, polysaccharopeptides, functional proteins (ubiquitin-like peptide, ubiquinone-9, glycoprotein, and nebrodeolysin), proteoglycans and glucans [35]. Proteins extracted from *Ganoderma. lucidum* and lectins, the sugar-binding proteins from edible mushrooms, have the capability to modulate the immune system of humans by stimulating (in vitro) the maturation of immune cells in the human immune system [111,122]. The immunomodulating effect of mushrooms resulting in the destruction of tumours has been reported by Guggenheim et al. [123].

Dietary white button mushrooms have been described to increase the movement of NK (natural killer) cells in mice. NK cells are a significant part of the immune system and are responsible for anti-tumour and anti-viral defence. The increased NK activity can be intervened by better production of IFN-g and TNF-a. The intake of *Agaricus bisporus* (white button mushrooms) resulted in a shift headed for T-helper 1 response, and there is a tendency for higher IL-2 and lymphocyte production [124].

Although mushrooms are highly nutritive and possess potential therapeutic benefits, there are some limitations, due to which these are not widely consumed by different sections of society. A sugar termed as ‘trehalose’ is extensively present in all edible mushrooms, and some people are allergic or intolerant to this sugar. This intolerance is caused by a dominant autosomic transmission alteration of trehalase, the enzyme responsible for the digestion of this sugar [125]. These have also been reported to increase the risk of Crohn’s disease, chronic inflammatory disease of the gastrointestinal tract [126]. Jin et al. [127] found that a lethal protein in *Agrocybe aegerita* was linked to hepatotoxicity due to the presence of a lectin in *A. aegerita*, which is resistant to the degradation by digestive enzymes in the human intestinal tract.

## 5. Processing Aspects of Edible Mushrooms

Edible mushrooms are easily vulnerable to browning reactions and other deteriorative changes, due to their high respiration rate, more enzymatic activity, and greater loss of water after harvesting. At ambient temperature conditions, these have a very short life, and remain acceptable only for a very short period. Therefore, the processing of edible mushrooms is highly commended to prevent spoilage and extension of its shelf life. Various reports on the processing of mushrooms as available in the literature are discussed in the following paragraphs.

Argyropoulos et al. [128] measured the effect of numerous drying techniques such as hot air, in combination with microwave vacuum drying, convective hot-air drying, and freeze drying on quality attributes of pre-treated mushrooms and found that combined drying by microwave vacuum and hot air produced a product with better quality as compared to drying by conventional methods such as hot air drying, showing higher porosity, lower overall color variation, softer texture, and greater rehydration ratio. Kulshreshtha and Singh [129] studied drying features, as well as the quality of the dried edible mushrooms dried by fluidized bed drying. They concluded that mushrooms dried at an air temperature of 50 degrees were better in quality, resulting in a product with better rehydration characteristics, reduced shrinkage, and brighter colour. Kumar and Barmanray [130] dried button mushrooms (*Agaricus bisporus* L.) slices by three methods, i.e., sun drying, mechanical drying, and microwave oven drying after different pre-treatments. They concluded that microwave and hot air drying resulted in better quality than sun-dried samples. Samples pre-treated with 0.5% potassium metabisulfite (KMS) solution + 2% citric acid solution after blanching in water produced superior quality products in comparison to other treatments.

Ratti, [131] reported that mushrooms can be dried using the microwave or freeze-drying technique, either independently or in combination with hot air drying. She further reported freeze-drying as an advanced drying method to preserve product quality. Krishna et al. [132] worked on the osmotic dehydration of white button mushroom exposed to osmotic dehydration at diverse concentrations (10, 15, 20, and 25%) of sodium chloride (common salt) and sugar solution (50, 60, and 70 °B), and further drying in a hot air oven at 55 ± 2 °C. They found better quality products after osmotic treatment with 70 °B sugar and 25% salt concentrations with low values for optical density. The products were then packed in polypropylene bags (200 gauge) and were found organoleptically acceptable and microbiologically safe for up to one year during storage at ambient conditions.

Lin et al. [133] treated button mushrooms with high CO_2_ (95–100%) during packaging, and found that treatment with CO_2_ for 12 h decreased the browning index to a significant level. High CO_2_ treatment augmented the antioxidant potential of mushrooms and retained the quality, flavour, and consumer acceptance of mushrooms. Farokhian et al. [134] evaluated the efficacy of ascorbic acid (40 g/L), chitosan (2%), cinnamon oil (50%), citric acid (40 g/L), lavender oil (70%), and heat processing techniques to extend the shelf-life of sliced edible mushrooms. Results concluded that treatment with lavender oil improved overall acceptability, browning index, weight loss, and marketability, as well as the firmness of mushrooms. The effects of different processing treatments on the nutritional quality of edible mushrooms are depicted in Table 2.

Robertson and Hoy [145] reported that the browning and softening processes related to the maturing of some vegetables and fruits, like mushrooms, can be postponed by the application of the irradiation technique. Therefore, irradiation, in combination with other innovative food processing techniques, can help to preserve nutrition, as well as extend the shelf life of edible mushrooms. Minnaar et al. [146] found that post-harvest treatments, like refrigeration (4 °C), along with gamma irradiation with a low dose, can increase the shelf-life of fresh edible mushrooms with the lowest quality losses.

Prevention of discolouration in fresh mushrooms was attempted by the application of radiation treatment. The lower intensity of browning was detected in irradiated samples as compared to control ones. Further, with increasing radiation dose up to 3 kGy, there was a decrease in browning during storage [147]. Xiong et al. [148] found that a dose of 1.2 kGy can delay browning, fruit body softening, and splitting in comparison to controls (non-irradiated) by 6–9 days. The effect of irradiation treatments on the composition of edible mushrooms is presented in Table 3.

Different varieties of mushrooms have been utilized for preparing value-added products, such as pickle [161], soup powder [162,163], chutney [164], bakery products like bread [165], biscuits [130,166] and its combination with tomato pulp for the preparation of tomato–mushroom-mixed ketchup [167] and tomato–mushroom-mixed soup [168]. Value-added products prepared from different varieties of edible mushrooms are represented in Table 4.

## 6. Conclusions and Future Aspects

The mushrooms have extensive potential to be used in the diet for taking advantage of the nutraceutical properties of the bioactive compounds. Edible mushrooms, due to lower fat and higher protein contents, need to be used for the preparation of low-calorie and high protein diets. Besides, their use as therapeutic foods can be helpful in the preclusion of lifestyle diseases, such as diabetes, hypertension, cancer, hypercholesterolemia, and cardiovascular diseases. Functional characteristics of these edible mushrooms are primarily due to the occurrence of antioxidants, dietary fibres, lectins, anti-microbial agents, and other bioactive components. Due to their richness in immune-modulating polysaccharides, these can be incorporated into health-promoting supplements. The approach for extraction of secondary metabolites from edible mushrooms is yet to be discovered. The exploration of new edible mushrooms and extraction of their valuable bioactive and their further incorporation for the preparation of value-added functional foods and their use in prevention, as well as the treatment of various lifestyle diseases, will continue to be the major attention of research into future prospects.

## Figures and Tables

**Table 1:** Bioactive compounds of edible mushrooms and their health benefits.

Mushroom (Common Name)	Bioactive Compounds	Health Benefits	References
*Agaricus bisporus* (White mushroom)	Pyrogallol, hydroxybenzoic acid derivatives, flavonoids, lectins	Anti-inflammatory, enhanced insulin secretion, anti-ageing property	[14,15,16]
*Auricularia auricular* (Jew’s ear mushroom)	Glucan, acidic polysaccharides	Immunomodulatory, anti-tumour, anti-inflammatory, lowers cholesterol and triglycerides, hypoglycaemic activity, immune tonic, and beneficial in coronary heart disease	[16,17]
*Flammulina velutipes* (Golden needle mushroom)	Peptidoglycan, polysaccharides, flammulin, FVP (flammulina polysaccharide-protein), proflamin (glycoprotein), a prolamin (active sugar protein)	Anti-inflammatory, antiviral, anti-tumour, antioxidant, activity, immuno-modulatory, anti-ageing property, anti-viral action	[16,18,19,20,21]
*Ganoderma lucidum* (Reishi, lingzhi)	Ganoderic acids, ganodermanontriol, ganoderiol, polysaccharides, germanium, triterpenoids, nucleotides and nucleosides, β-glucan	Anti-metastatic, anti-tumour, anti-viral, anti-HIV, immunomodulatory, antibiotic properties, liver protection, prevents cholesterol synthesis	[22,23]
*Lentinula edodes* (Shiitake)	Lentinan, glucan, mannoglucan, fucomannogalactan, lentin (protein), catechinflavonoids, eritadenine	Immunomodulatory, anti-tumour, anti-inflammatory, anti-fungal, antioxidant, anti-bacterial, antifungal, antioxidant, hypolipidemic activity	[24,25,26,27,28]
*Cordyceps sinensis* (Caterpillar fungus)	Cordycepin	treat lung infection, hypo-glycemic activity, cellular health properties, antidepressant activity	[16]
*Pleurotus florida* (White oyster)	β-glucans	Antioxidant, anti-microbial	[29,30]
*Pleurotus ostreatus* (Oyster mushroom)	Functional proteins (ubiquinone-9, ubiquitin-like peptide, nebrodeolysin, and glycoprotein), proteoglycans pleuran (β-1, 3-glucan with galactose, and mannose), glucans, proteoglycan, laccase, pleurostrin (peptide)	Immunomodulatory, hyperglycemia, anti-tumour, antioxidant, anti-viral, anti-fungal	[31,32,33,34,35]
*Grifola frondosa* (Ram’s head)	Lectins, polysaccharides	Decrease blood glucose improves insulin secretion and ovulation	[16]
*Pleurotus pulmonarius* (Lung oyster mushroom)	Polysaccharides such as β (1,3)-glucopyranosyl, and Polysaccharides (1,3), (1,6)-linked β-glucan	Anti-inflammatory	[36,37]
*Volvariella volvacea* (Paddy straw mushroom)	Fip-vvo	Immunomodulatory	[38]
*Hericiumerinaceus* (Monkey head mushroom)	Hericenones and erinacines	Neuritogenic effects	[16]

**Table 2:** Effects of different processing techniques on the nutritional composition of mushrooms.

Mushrooms	Methods of Processing/Storage	Effect on Nutritional Composition	References
*Agaricus bisporus*	Freezing at −25 °C, canning and salting for 6 months	The protein content was reduced to 24.3 percent, 22.2 percent, 16.54 percent, in canning, freezing, and salting respectively; decrease in free amino acids (cysteine, tyrosine, glutamine, alanine) in all treatments.	[135]
	Blanching at 95–100 °C for 15 min	Decreased levels of minerals	[136]
	Stored at 12 °C for 12 days	The decrease in sugar content, fructose, and mannitol; increase in free amino acids from 77.92 to 140.57 g/kg	[137]
*Macrolepiota procera*	Freezing, drying, and gamma irradiation	Higher DPPH scavenging activity was reported in dried samples while freeze and irradiated samples showed higher reducing power	[138]
*P. ostreatus*	Oven-dried at 60 °C till a constant weight obtained, Blanching at 88 °C for 1 min, Brining (25% salt solution) for 30–60 min	The protein content decreased and carbohydrates get enhanced during oven drying. It was also observed that protein, fat, and carbohydrate contents get reduced during blanching and brining	[139]
	Freezer storage for 12 months	The decrease in some amino acids such as alanine, glycine, histidine, threonine serine, and methionine)	[140]
	Microwave processing and frying	Reduction in the amount of Fe, Zn, Mn, Ca, and Cu during microwave processing and increase in Iron content during frying	[141]
*Macrolepiota mastoidea, Lactarius deliciosus,* *Sarcodon imbricatus*, and *Macrolepiota procera*	Drying, freezing, and cooking	Antioxidant activities and nutrient concentrations of cooked samples was lower than either of dried or frozen mushroom samples	[142]
*Lentinus edodes*	Heat treatment	There was a significant increase in DPPH and ABTS radical scavenging activities by 2.2-fold and 2.0-fold, respectively as compared to the raw sample	[143]
*Amanita zambiana*	Frying, microwave heating, boiling, drying	Frying increased proteins, lipids, and carbohydrates, microwave heating increased the proteins and carbohydrates content while boiling only increased the carbohydrate content and decreased the phenolic contents, drying increased the proteins, carbohydrates, and total phenolic components	[144]
*Lentinula edodes, Agaricus bisporus, Pleurotus eryngii*, and *Pleurotus ostreatus*	Boiling, microwaving, grilling, and deep-frying	Significant loss of ash, carbohydrates and protein contents, during frying but increase in energy as well as fat contents. Further, boiling enhanced the total glucan contents decreased the antioxidant activity significant especially after frying and, boiling as compared to microwaved and grilled mushrooms	[4]

**Table 3:** Effect of irradiation treatments on the composition of edible mushrooms.

Edible Fungi	Treatment Conditions	Major Findings	References
Fresh shiitake mushrooms, oyster mushroom, button mushroom, and abalone mushroom	Ultra Violet-A (wavelength 315 to 400 nm) Ultra Violet-B (wavelength 290 to 315 nm) Ultra Violet-C (wavelength 190 to 290 nm) for 1 h	Increased amounts of vitamin D2 content	[149]
Six species from genus *Agaricus, Auricularia*, *Agrocybe, Lentinula, Hypsizigus,* and *Pholiota*, and five species from *Pleurotus genus*	Ultra Violet-B for 2 h	Increase in Vitamin D2 content and antioxidant activity	[150]
*Macrolepiota prolera*	γ-Irradiation (0.5 and 1 kGy)	The freezing and over-drying were attenuated by irradiation treatment	[151]
*Pleurotus ferulae*	Pulsed irradiation (19 to 700 nm; 60 pulses)	Increase in vitamin D2 and bone density of PM mice with increased osteoblast and lower osteoclast cells	[152]
*Lentinula edodes*	γ-Irradiation (1 kGy)	Increase in phenolic compounds and antioxidant activity of mushroom	[153]
*Agaricus bisporus*	γ-irradiation	Increased total phenolic components and phenylalanine ammonia-lyase activity	[154]
	γ-Irradiation (1, 3, and 5 kGy)	Irradiation significantly reduced the concentration of guanosine 5′-diphosphate (22%) and adenosine 5′-monophosphate (AMP) (46%).	[155]
*Pleurotus ostreatus*	^60^Co γ-Irradiation	Irradiation treatment increased phenolic content, flavonoids, and antioxidant activity of dried mushroom	[156]
	Mushrooms were illuminated with UV-B with a light intensity of 310–320 nm and 11.5 W/m^2^ for 60 min at 20 °C	The accumulation of vitamin D2 > 100 μg. The concentration of Photo-products such as lumisterol, tachysterol, and pre-vitamin D2 increased concurrently.	[157]
	γ-Irradiation	The increased antioxidant potential, hygienic quality and extended shelf-life	[156]
	γ-Irradiation	Irradiation with 1 to 6 kGy as physical stress factors increased protein, carbohydrates, and glucans	[158]
	UV-B radiation	Increase in vitamin D2 content in irradiated mycelia of golden and pink oyster mushrooms as 0.28–5.93 and 66.03–81.71 μg/g, respectively.	[150]
*Pleurotus florida*	Photo-irradiation	Extracellular synthesis of silver nanoparticles from aqueous extract of the mushroom	[159]
		Synthesis of biofunctionalized gold nanoparticles	[160]

**Table 4:** Value-added products from edible mushrooms.

Mushroom	Products	References
*Pleurotus ostreatus*	Value-added products (biscuits, soups, pickles, jam, snacks)	[169]
	Butter biscuits, biscuits	[166,170,171]
	Cake	[172]
	Bread	[165,173,174]
	Potato Puddings	[175]
	Seasoning	[176]
*Agaricus bisporus*	Soup powder	[162,163]
	Drying	[128,177]
	Pickle	[161]
	Chutney	[164]
	Biscuits	[130,178]
	Ketchup	[167]
	Meat analogue	[179]
*Llentinus edodes*	Biscuits	[180]
	Bread	[181]
	Muffin	[182]
	Seasoning	[183]
	Brown sauce	[184]
*Ganoderma lucidum*	Functional bread	[185]
	Drink (Beer, Yakju)	[186]
*Pleurotus plumonarius*	Bread	[165]
*Pleurotus sajor-caju*	Flat bread, rice-porridge and conventional cake	[187]
	Biscuits	[171]
*Pleurotus eryngii*	Sponge cake	[188]

## Data Availability

Data sharing not applicable.
